# Simple Methods and Rational Design for Enhancing Aptamer Sensitivity and Specificity

**DOI:** 10.3389/fmolb.2018.00041

**Published:** 2018-05-14

**Authors:** Priya Kalra, Abhijeet Dhiman, William C. Cho, John G. Bruno, Tarun K. Sharma

**Affiliations:** ^1^Department of Biotechnology, All India Institute of Medical Sciences, New Delhi, India; ^2^Faculty of Pharmacy, Uttarakhand Technical University, Dehradun, India; ^3^Department of Clinical Oncology, Queen Elizabeth Hospital, Kowloon, Hong Kong; ^4^Operational Technologies Corporation, San Antonio, TX, United States; ^5^Center for Biodesign and Diagnostics, Translational Health Science and Technology Institute, Faridabad, India; ^6^AptaBharat Innovation Private Limited, Translational Health Science and Technology Institute Incubator, Faridabad, India

**Keywords:** aptamers, SELEX, affinity, K_d_, selectivity, specificity, limit of detection, sensitivity

## Abstract

Aptamers are structured nucleic acid molecules that can bind to their targets with high affinity and specificity. However, conventional SELEX (Systematic Evolution of Ligands by EXponential enrichment) methods may not necessarily produce aptamers of desired affinity and specificity. Thus, to address these questions, this perspective is intended to suggest some approaches and tips along with novel selection methods to enhance evolution of aptamers. This perspective covers latest novel innovations as well as a broad range of well-established approaches to improve the individual binding parameters (aptamer affinity, avidity, specificity and/or selectivity) of aptamers during and/or post-SELEX. The advantages and limitations of individual aptamer selection methods and post-SELEX optimizations, along with rational approaches to overcome these limitations are elucidated in each case. Further the impact of chosen selection milieus, linker-systems, aptamer cocktails and detection modules utilized in conjunction with target-specific aptamers, on the overall assay performance are discussed in detail, each with its own advantages and limitations. The simple variations suggested are easily available for facile implementation during and/or post-SELEX to develop ultrasensitive and specific assays. Finally, success studies of established aptamer-based assays are discussed, highlighting how they utilized some of the suggested methodologies to develop commercially successful point-of-care diagnostic assays.

## Introduction

Aptamers recognize and bind to their targets through 3-dimensional shapes and various physiochemical interactions, similar to antibody binding mechanisms. These interactions include, but are not limited to, hydrophobic, electrostatic, hydrogen bonding, van der Waals forces, base stacking, and shape complementarity. These functional nucleic acid molecules or aptamers owing to their flexibility, small size, and reduced steric hindrance can recognize biomolecules with ease, offering vast potential in diagnostics, therapeutics or drug-delivery systems (Catuogno et al., 2016; Sharma et al., 2016; Dhiman et al., 2017; Zhou and Rossi, 2017). In particular, aptamer-based biosensing systems have been widely used for detection of proteins, chemicals, and metabolites in biological samples, environmental substances, and food products (Sharma et al., 2014; Weerathunge et al., 2014; Zhou et al., 2014). Regardless of the downstream application, it is necessary for aptamers to demonstrate high affinity, avidity, specificity and/or selectivity since it ultimately dictates the formulation of successful sensitive and specific assay. It must be kept in mind that both affinity and specificity are important parameters central to any diagnostic and/or therapeutic application. Although, several researchers have worked hard to understand the correlation between affinity and specificity, they have not been able to draw any strong correlation between them. Thus, in the current perspective we have attempted to discuss new innovations in SELEX with few pragmatic suggestions to enhance aptamer affinity and/or specificity that are not difficult to implement as most of these methods are readily available for routine implementation in aptamer development laboratories.

## Enhancing aptamer evaluation parameters

It is logical to interpret that any conformationally flexible aptamer showing “induced-fit” strategy of binding would also demonstrate interaction with many off-targets having similar motifs, structural similarity etc. In contrast, molecules with defined ground state, would bind only to their specific targets with high affinity. Although underrated, conformational flexibility is a critical factor dictating the affinity and specificity of aptamer-target interactions. It is therefore important to understand the binding interactions between an aptamer and target, as they would ultimately help to rationalize and improve aptamer structures for downstream applications (Eaton et al., 1995). Understanding the binding parameters such as affinity, specificity, selectivity etc. assesses these interactions.

### Affinity

The term affinity refers to the strength of interaction that may exist between an aptamer and its target and is often assessed by measuring the binding or association constant (K_a_), which is inversely proportional to the dissociation constant (K_d_). In the kinetics of interaction of aptamer [A] with target [T]: A + T ⇌ AT; the association constant (K_a_) at equilibrium is the ratio of on-rate (*k*_*forward*_) and off-rate (*k*_*reverse*_) constants.

(1) $$\begin{array}{l} {\text{K}}_{\text{a}}=\frac{1}{{\text{K}}_{\text{d}}}=\frac{\text{on}-\text{rate}}{\text{off}-\text{rate}}=\frac{k_{forward}}{k_{reverse}} \end{array}$$

Oligonucleotides that have slow-off rates or low dissociation constants exhibit strong interactions with their target. As a result, these high affinity aptamers can bind even low amounts of target in samples (low limit of detection), making them sensitive affinity reagents. This is in particular useful in the discovery of diagnostic biomarkers that are occasionally low in abundance in body fluids. It is logical to assume that increasing the number and nature of interactions of aptamers with target, would allow evolution of slower off-rate or higher affinity aptamers (Hasegawa et al., 2016). But the limited chemical diversity of traditional DNA/RNA libraries (four nucleotides) is perhaps the most obvious shortcoming in traditional SELEX to evolve high affinity aptamers. On the other hand, their proteinaceous counterpart antibodies can mix and mingle 20 different hydrophilic and hydrophobic amino acids to diversify their binding sites. Moreover, due to a hydrophilic polyanionic backbone, traditional aptamers lack or exhibit weak hydrophobic interactions limiting their functional contacts and binding properties with the target. To counter this deficiency, new SELEX derivative methods (Table 1) such as X-aptamers from Gorenstein's laboratory (He et al., 2012) and Xeno Nucleic Acids or XNAs (Pinheiro and Holliger, 2014) including SOMAmers (Gold et al., 2010), LNAs (Hernandez et al., 2009) etc., are a subject of intense study for their ability to enhance aptamer affinity, by utilizing modified nucleotides that do not occur in nature.

**Table 1:** Innovations to generate high affinity aptamers.

**S.No**.	**Innovations**	**Examples**
1.	**Xeno Nucleic Acids (XNAs)** *(During or Post-SELEX)*	Part of artificially expanded genetic information system (AEGIS); Synthetic alternate to natural nucleic acids DNA and RNA; function as biopolymers with applications in xenobiology (Pinheiro and Holliger, 2014; Lipi et al., 2016). AEGIS-SELEX evolved XNA aptamer with non-standard P,Z nucleotides showing high affinity (nM K_d_) toward breast cancer cell line (Sefah et al., 2014).
	**Slow-Off rate Modified Aptamers (SOMAmers)** (*Protein-like modifications*)	13 “difficult” human proteins that failed unmodified DNA SELEX, yielded high affinity (nM K_d_) slow off-rate aptamers using modified DNA libraries (Gold et al., 2010). High affinity ultrasensitive detection of analytes with 1 pM−50 fM limit of detection (Brody et al., 2010).
	**Locked Nucleic Acids (LNAs)** (*Locked sugar rings with 2′O, 4′C methylene linkage*)	Replacement of purine nucleotides in the avidin-binding aptamer with LNAs, enhanced aptamer binding affinity by 8.5 folds (Hernandez et al., 2009).
	**Chemical modifications** (*Modifications in base, sugar or phosphate backbone, during or Post-SELEX*)	5′-BzdU modified nucleolin AS1411 aptamer demonstrated higher binding affinity to cancerous cells than unmodified aptamer (Lee et al., 2010). Phosphorodithioate (PS2) substitution on single nucleotide of α-thrombin specific RNA aptamer enhances binding affinity by ~1000 folds (Zandarashvili et al., 2015; Abeydeera et al., 2016).
2.	**Multimerization** (*Enhances avidity; Applicable during or Post-SELEX*)	A bivalent aptamer constructed by connecting two identical 3R02 aptamers (300 pM Kd) via a 10-mer thymine linker demonstrated 10 folds higher binding (30 pM K_d_) to VEGF than the monomer (Nonaka et al., 2013). Joining two established α-thrombin specific 15-mer and 29-mer aptamers using a flexible PEG-phosphoramidite linker, enhanced binding affinity of bivalent aptamer by 97 folds over, the individual monomers (Tian and Heyduk, 2009). Bivalent aptamer having two 15-mer thrombin specific aptamers joined by a 35-mer random region from DNA library was isolated by evolution strategy and showed >200 folds higher affinity (<10 pM K_d_) than the 15-mer monomer (Ahmad et al., 2012).
3.	**Truncations and mutations** (*Post-SELEX*)	Truncated 14 nt transferrin receptor specific aptamer demonstrated 6 folds higher affinity than the 50 nt parent aptamer (Macdonald et al., 2016). Truncated VEa5 aptamer exhibits >200 fold higher binding affinity to VEGF (Kaur and Yung, 2012). *In silico* maturation (ISM) based mutagenesis successfully improved binding affinity of VEGF specific 3R02 aptamer 16 folds higher than the parent VEap121 aptamer (Nonaka et al., 2013).
4.	**X-aptamers** (*Non-SELEX approach*)	Utilizes bead-based selection approach, bypasses the need for enzyme amplification, therefore shows high compatibility with unlimited functional groups to generate X-aptamers of nM affinity in a single round (He et al., 2012; Lam et al., 2015).
5.	**Non-equilibrium Capillary Electrophoresis of Equilibrium Mixtures (NECEEM)** (*Non-SELEX approach*)	NECEEM efficiently partitions aptamer-target complexes based on binding parameters (K_d_, *k_*on*_, k_*off*_*) leading to one-step evolution of high affinity (nM K_d_) aptamers (Berezovski et al., 2006).
6.	**Magnetic-assisted rapid aptamer selection (MARAS)** (*Non-SELEX approach*)	MARAS protocol utilizes magnetic beads and optimized amounts of externally applied rotating magnetic fields for selection of ultrasensitive aptamers having low nM K_d_, without the need for iterative selection. Method is also compatible with primer-free libraries unlike conventional SELEX (Lai and Hong, 2014; Tsao et al., 2017).
7.	**Quantitative Selection of Aptamers through Sequencing (QSAS)** (*M-SELEX + high throughput sequencing*)	Cho et al. (2010) utilized QSAS method to simultaneously track and enrich >10 million individual aptamers through selection to identify high affinity aptamers within 3 rounds. QSAS evolved aptamers evinced ~3-8 folds higher affinity than traditional SELEX evolved aptamers.
8.	***In silico*** **evolution approaches** (*Non-SELEX; in silico enrichment*)	Utilization of Bioinformatics tools in combination with established *in vitro* techniques for *in silico* enrichment of aptamer sequences.
	**Closed Loop Aptameric Directed Evolution (CLADE)**	Combines microarray approach with the identification and evolution of sequences by *in silico* methodology, successful in identification of nM affinity aptamers (Knight et al., 2009).
	**Aptamer affinity maturation**	Enriched sequences with aptamer families are utilized to evolve aptamer motifs, which were screened by Microarray to evolve ~10-folds higher affinity PfLDH aptamers (Kinghorn et al., 2016).

XNA-based aptamers displaying superior binding properties have successfully been generated through myriad modifications of phosphate-backbone, nucleotide bases and sugar-rings, in the random- as well as the primer-binding region of oligonucleotides (Lipi et al., 2016). While incorporation of these beneficial modifications may also be done in identified aptamer candidates post-SELEX, nevertheless screenings have to be repeated to assess relative improvement in binding affinities. Which is why incorporation of modified nucleotides in the starting libraries is beneficial, as it allows evolution to select target specific high affinity aptamers through SELEX. However, these “unnatural” or synthetic nucleotides stall the enrichment and sequencing steps in SELEX, due to poor enzyme recognition capabilities.

A successful example of XNAs includes the Slow-Off rate Modified Aptamers (SOMAmer), that as the name suggests are base-modified by giving aptamers protein-like functionality that have enhanced hydrophobic interactions, slow-off rates, and therefore high affinity (Davies et al., 2012). These SOMAmer reagents, addressed as next generation aptamers, on an average demonstrate ultrasensitive limit of analyte detection from 1 pM to as low as 50 fM (Brody et al., 2010). In the area of modified base aptamers, the most recent radical development is that of “Seligos” produced by Apta Biosciences, Ltd. of Singapore and the United Kingdom (a recent spinoff of Fujitsu Laboratories). In Seligos, pendant amino acids extending from a nucleic acid backbone effectively imitate natural peptides or proteins, enhancing overall binding parameters (Fujita et al., 2012).

Yet another apt example to state is the study conducted by Gold et al. (2010) who demonstrated the utility of chemically modified aptamers in high throughput multiplexed proteomics technology by discovering 58 potential chronic kidney disease biomarkers in patient serum samples. The chemical modification of nucleotides enhanced the overall aptamer performance several folds to enable a median 1 pM limit of detection of serum biomarkers. Using this method, Gold et al., have succeeded in coalescing high sensitivity and specificity in a multiplex assay (Wilson, 2013). As a part of Artificially Expanded Genetic Information System (AEGIS) and genetic alphabet expansion SELEX (ExSELEX), using “unnatural” or synthetic nucleotides, the evolved XNA-aptamers demonstrate low nM to pM range dissociation constants and therefore ultrahigh affinity (Sefah et al., 2014; Biondi et al., 2016; Kimoto et al., 2016). However this approach heavily relies on the ability of polymerases to accept modified or artificial nucleotide triphosphates as substrates and their ability to “deep sequence” these XNA aptamers (Yang et al., 2011; Sefah et al., 2014; Biondi et al., 2016). Although several groups have now developed engineered enzymes to support XNA-SELEX (Loakes and Holliger, 2009; Siegmund et al., 2012; Kasahara et al., 2013; Aschenbrenner and Marx, 2016; Larsen et al., 2016; Wang et al., 2016), many nucleotide-modifications with favorable chemistries such as positive charge, hydrophobic groups, phosphorothioates, amino acids etc. are missed as they are difficult or even impossible to amplify and sequence.

To circumvent these problems, a non-SELEX method of X-aptamers uses the “one bead-one modified aptamer” selection approach that bypasses the need of enrichment steps in aptamer evolution; and therefore, supports incorporation of myriad modified nucleotides for enhanced diversity. X-aptamers have successfully been shown to increase binding affinity toward target proteins by at least 23-fold in single step (He et al., 2012). X-aptamers are also modified to have “thio” or phosphorothioate backbone having oxygen replaced with sulfur in the phosphodiester bonds, to better resist serum nucleases. Another successful method of obtaining high affinity aptamers includes Non-equilibrium capillary electrophoresis of equilibrium mixtures (NECEEM). This non-SELEX method is based on affinity-partitioning that simultaneously monitors the bulk affinity of enriched libraries along with the affinity of clones at each step. Since NECEEM is rapid and surpasses the need for multiple SELEX-rounds, it enables identification of high affinity nanomolar K_d_ aptamers in a single round (Berezovski et al., 2006). Another efficient partitioning-based novel method described by Lai and Hong (2014) utilizes magnetic beads. Application of externally applied rotating magnetic fields of optimized amplitude/frequency is used to partition target specific aptamers of desirable affinity. Termed as Magnetic-Assisted Rapid Aptamer Selection (MARAS) target-specific nanomolar affinity aptamers are easy to isolate rapidly, without the need for multiple selection rounds, thereby also encouraging utility of primer-binding domain free libraries (Tsao et al., 2017). It is therefore safe to anticipate, X-aptamer methodology or the amalgamation of Xeno Nucleic Acid chemistry with the affinity-partition based NECEEM or MARAS approach would provide one-step selection of ultrasensitive aptamers displaying robust binding. However, it is imperative to understand that innovations introduced during or post-SELEX have their own respective pros and cons. “During SELEX” strategies on one hand allow natural evolution of high affinity aptamers, they may require specialized technology and engineered enzymes for incorporation of exotic-nucleotides. On the other hand, although Post-SELEX strategies bypass the requirement of specialized enzymes for amplification, however, re-screening of the generated modified aptamers is strongly encouraged, as these diversifications may drastically enhance or even reduce aptamer binding parameters.

In addition, widely available molecular modeling software programs such as PatchDock and YASARA following generation of PDB files and dot-bracket notations in RNA Composer (Popenda et al., 2012; Heiat et al., 2016; Bruno, 2017a) or other free on line programs or web servers can be used to generate useful 3-dimensional docking models. These rotatable 3-D models can help aptamer developers to better understand their putative binding sites and to manipulate them with insertion of exotic unnatural bases as a post-SELEX approach. Bruno (2015b) used a similar 2-D approach with UNAFold software to predict and manipulate an aptamer binding site to obtain greater affinity for a *Rickettsia typhi* cell binding aptamer by incorporation of several diaminopurines (DAPs) into the probable binding loop. Accordingly, Gawande et al. (2017) at SomaLogic, Inc. have recently demonstrated enhanced affinity and greater inhibition of a convertase by engineering two amino acid-like modified nucleotides into the binding site of a SOMAmer (aptamer).

Toward the end of SELEX, only a handful of cloned aptamers are randomly picked and characterized, as a result majority of final pool, which may contain several beneficial ultrasensitive sequences may be unintentionally lost. Therefore, researchers are now dedicated to identify combinatorial technologies, that provide maximum resolution and sequence identification. A successful example is of a novel methodology Quantitative Selection of Aptamers through Sequencing (QSAS) described by Cho et al. (2010), that evolves unmodified aptamers of high affinity than conventional selection methods. QSAS combines the power of Microfluidics technology (M-SELEX), that shows accelerated generation of high affinity aptamers (Lou et al., 2009; Oh et al., 2009), with the high throughput sequencing technology (Zimmermann et al., 2010), to evolve unmodified aptamers with ~3–8-folds higher affinity than the conventional SELEX. Although effective and fast, implementation of this technology is heavily dependent on sophisticated instrumentation and tools. Additionally not all technology combinations with SELEX improve the evolution of high performance aptamers e.g., point mutation analysis (Platt et al., 2009); microarray technology (Katilius et al., 2007) etc. However, a non-SELEX approach combining microarray technology with the *in silico* Closed Loop Aptameric Directed Evolution (CLADE) showed promising success in obtaining aptamers of highest (nM K_d_) binding parameters (Knight et al., 2009). Another novel *in silico “*aptamer affinity maturation” approach, analogous to the antibody affinity maturation process was described by Kinghorn et al. (2016). The method employs generation of aptamer family motif from the aptamer library containing the best possible aptamer sequence, from the original SELEX data. Thereafter using a DNA microarray, the aptamer motif created library was massively screened against malaria PfLDH to identify resampled aptamers with ~10-folds higher affinity (Kinghorn et al., 2016), than their previously identified 2008s aptamer (Cheung et al., 2013). Although effective and fast, implementation of these combinatorial and evolutionary computation technologies is at times costly with heavy dependency on sophisticated instrumentation or the availability of bioinformatics tools and technological expertise.

Several simple and easy-to-implement moderations can be introduced in conventional SELEX to ensure evolution of aptamers of best characteristics. For instance, a preliminary factor that may dictate the affinity of an aptamer pool is the number of SELEX rounds. As a practical matter based on our experience, it is advisable to select and amplify aptamers until the candidate aptamer pool no longer elutes from the immobilized target at 99°C in nuclease free water, as demonstrated by the lack of an aptamer PCR amplicon band following electrophoresis. The developer can then go back to the previous SELEX round which still produced an aptamer band on an electrophoresis gel following PCR of the eluted aptamer, for cloning in *E. coli* and DNA sequencing to obtain the highest possible affinity aptamer pool. The presence of high affinity aptamers in this last elutable aptamer pool can be verified by enzyme-linked aptamer sorbent assay (ELASA) and/or surface plasmon resonance (SPR), for screening and ranking of the candidate aptamers. Continual gel purification of the aptamer amplicon band of the correct length by excision and elution of gel slices will typically result in enhanced aptamer purity and therefore greater affinity and assay sensitivity as well. If aptamers tend to appear as smeared products (likely concatemers) on gel, the developer can either cut and elute only the correct length DNA or RNA, especially from a denaturing urea-based electrophoresis gel, decrease the number of PCR cycles or add *E. coli* single-strand binding protein (SSB), which is commercially available as Perfect Match^TM^ from Agilent Technologies, Inc. during PCR (Crameri and Stemmer, 1993).

**Figure 1:**
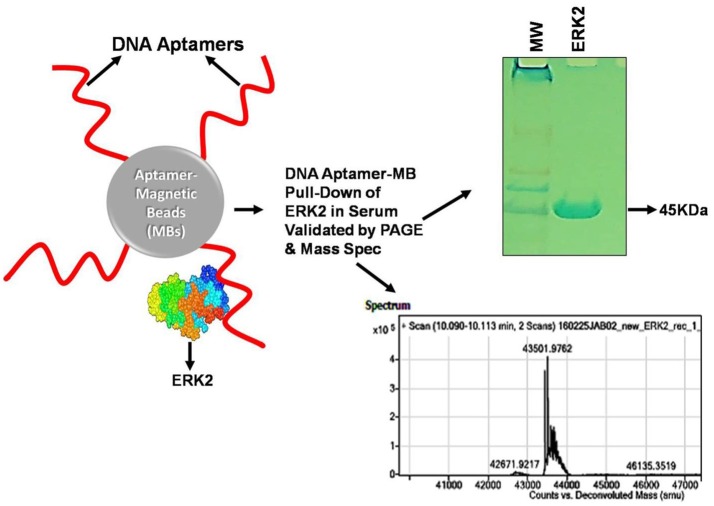
Illustration of a cancer biomarker (ERK2) aptamer pull-down assay on aptamer-coated magnetic beads (MBs) performed in serum and analyzed by gel electrophoresis and mass spectrometry. The “clean” ~43.5 kD ERK2 band shown in the gel and clear mass spectral signature virtually free of interfering signals from serum were likely due to original aptamer selection in diluted serum as well as the addition of 2 mM EDTA to chelate free calcium ions, thereby enhancing aptamer specificity (Bruno, 2017b). Anti-ERK2 aptamers are shown as wavy red lines attached to MBs either covalently or via streptavidin-biotin linkages which then capture any free extant ERK2 or spiked recombinant ERK2 (rERK2) in serum samples. Following MB collection with a strong permanent magnet (pull-down), removal of serum, several buffer washes and elution of the ERK2 from the aptamer MBs in strong (0.1 M) HCl, followed by removal of the aptamer-MBs and neutralization of ERK2 in the acid with 0.1 M NaOH, the resultant eluate is electrophoresed in polyacrylamide and analyzed by mass spectrometry to validate its molecular weight (MW) and probable identity. A real experimental Coomassie Blue-stained 12% polyacrylamide gel and mass spectrum are shown from experimental pull-down assay runs.

At the outset of any SELEX development program, it is wise to select aptamers in their intended chemical milieus and physical conditions to ultimately produce assays or therapeutics that are likely to function with high affinity and specificity in such environments. As a prime example, one of the author's (Bruno's) recent aptamer development projects for detection of the cancer biomarker (ERK2) resulted in “clean” aptamer-magnetic bead pull-down of ERK2 from human serum as confirmed by gel electrophoresis and mass spectral analysis (Figure 1), because the aptamer was selected in diluted serum (Bruno, 2017b). Furthermore, it is well known that ionic strength especially due to divalent cations such as Ca^2+^ and Mg^2+^ can greatly impact aptamer performance (Bruno et al., 2011b; Jeong and Rhee Paeng, 2012). However in contradiction, high ionic strength has also been reported to reduce the sensitivity of both 76mer-DNA and 57mer-RNA aptamers for tetracycline (Jeong and Rhee Paeng, 2012). Also, despite these aptamers having better affinity and specificity than the corresponding antibodies, the authors reported comparable sensitivity and specificity between aptamer-based enzyme-linked assays and the conventional antibody based ELISA assay (Jeong and Rhee Paeng, 2012).

Physical factors such as temperature should also be considered during SELEX to obtain aptamers of desired affinity. Although temperature is not necessarily a factor in aptamer performance, one should always select diagnostic aptamers at room temperature (20–25°C) and therapeutic aptamers at body temperature (37°C) to ensure the best results for final application. Two-dimensional computer modeling with Mfold or UNAfold as a function of temperature, will reveal or predict any temperature-related conformational changes in the stem-loop structures of aptamers that could affect aptamer affinity or specificity (Bruno, 2016).

Despite employing strategies to enhance high affinity aptamers *in vitro*, the selected aptamers may at times be in ineffective *in vivo*. This disparity may be due to accessibility or protection of target; *in vivo* conformation, half-life and bioavailability of aptamer. It is also possible that the intracellular environment affects the target conformation, which may change *in vivo*, thereby altering aptamer efficacy *in vivo* (Mi et al., 2010; Catuogno and Esposito, 2017). Thus to mimic the tissue microenvironment, *in vitro* 3D Cell-SELEX strategy (Souza et al., 2016) and *in vivo* SELEX strategy (Mi et al., 2010; Cheng et al., 2013) have been utilized by several groups to generate high affinity (nM K_d_) aptamers applicable *in vivo*, toward tumor markers or as delivery vehicles across blood-brain barrier. Additionally, these strategies allow aptamer selection in the physiological environment along with direct elimination of sequences of no interest, thereby providing an added advantage. One emerging promising application of aptamers *in vivo* is their role as biosensors that can regulate artificial riboswitches. These molecules possess a ligand sensing (aptamer) domain and an expression domain (regulatory riboswitch). The major concern in evolving RNA aptamer riboswitches is the discrepancy between *in vitro* binding and intracellular functional activity. While *in vivo* selection strategies maybe able to evolve cell-capable RNA aptamers, they suffer limitation in applicability as sensors (Berens et al., 2015). To circumvent this problem, Porter et al. (2017) have recently demonstrated the utility of secondary and tertiary structural scaffolds derived from naturally occurring ribozymes and riboswitches to evolve functional aptamers against small molecule precursors of neurotransmitters, that effectively function as biosensors *in vitro* as well as in the cellular context. Identification and incorporation of natural riboswitches with aptamers, is therefore a subject of intense focus amongst researchers.

**Figure 2:**
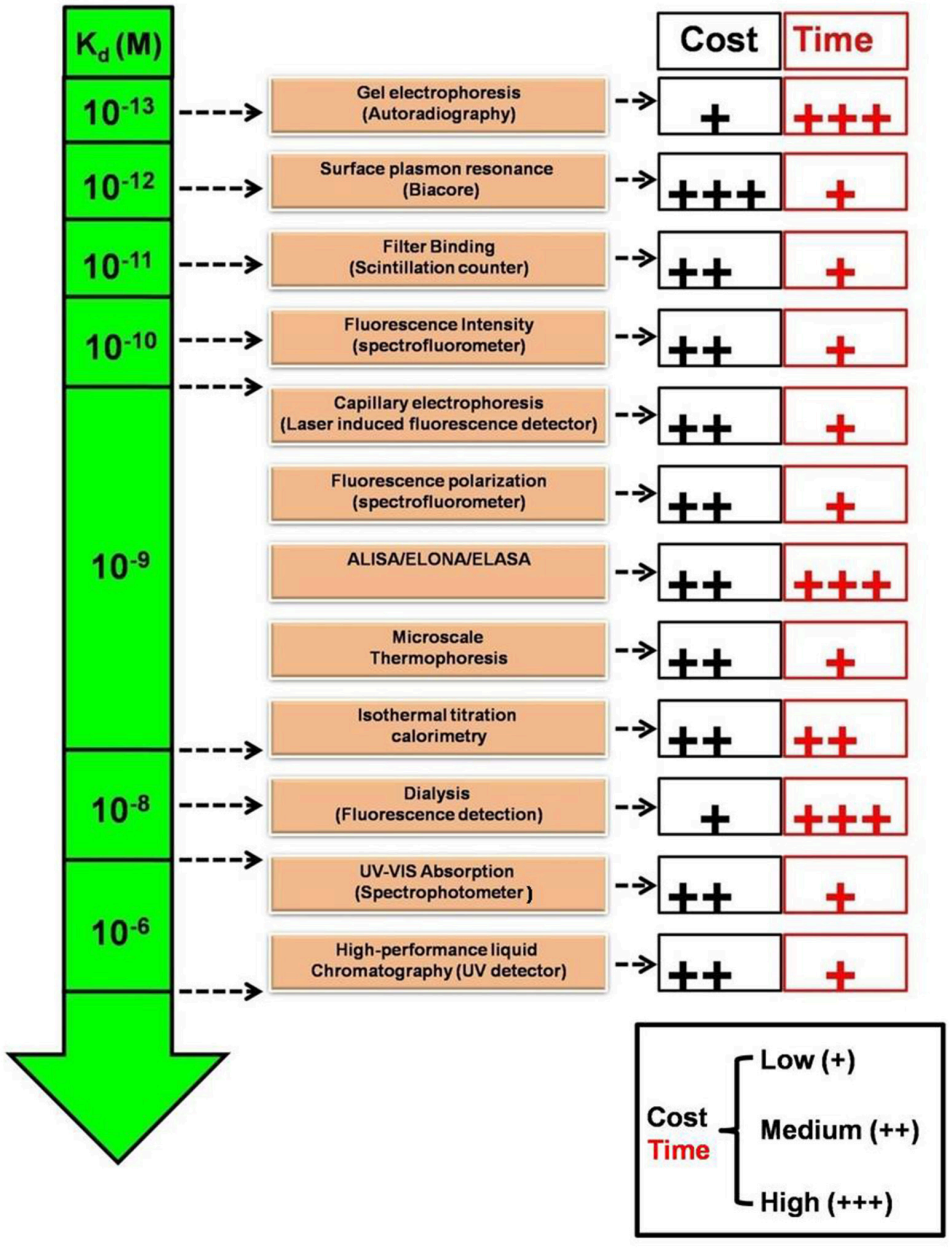
Comparison of relative sensitivities, turnaround time and cost of various K_d_ estimation techniques. K_d_, Apparent dissociation constant (Molar); UV-Vis, Ultraviolet-Visible; ALISA, Aptamer-linked immobilized sorbent assay; ELONA, Enzyme-linked oligonucleotide sorbent assay; ELASA, Enzyme-linked aptamer sorbent assay.

As described, one means to evaluate aptamer-target binding is by the measurement of binding constants. This measurement may also be relative in a sense; because it dramatically depends on the detection limit or sensitivity of the technique employed for estimation. Jing and Bowser (2011) and Gopinath et al. (2016) have discussed in detail the limitations and variations in different techniques available with the ranges of estimated apparent dissociation constant, K_d_, (Figure 2). While highly sensitive techniques employing radioactivity and SPR can measure K_d_ up to sub-picomolar range; Fluorescence and ITC can measure K_d_ limited to nanomolar and spectrophotometry techniques can measure K_d_ in micromolar range. Recently, MicroScale Thermophoresis (MST), a novel low cost highly sensitive technique has been described by numerous aptamer—research groups that can estimate apparent dissociation constant in pico to nanomolar range, with high accuracy in limited microliter scale solutions (Stoltenburg et al., 2015; Breitsprecher et al., 2016; Entzian and Schubert, 2016; Jauset Rubio et al., 2016). In addition to less volume and unlike several techniques, MST also offers the advantage, that it can estimate the aptamer K_d_ independent of the target size in wide range of buffers as well as in complex biological samples, in pico to nanomolar range (Entzian and Schubert, 2016). Given the variation in the estimated K_d_ that may arise technique to technique, it is imperative, that when comparing performance of aptamers relatively, K_d_ for all the candidates should be estimated by the same technique, using the same range of aptamer concentrations.

### Avidity

**Figure 3:**
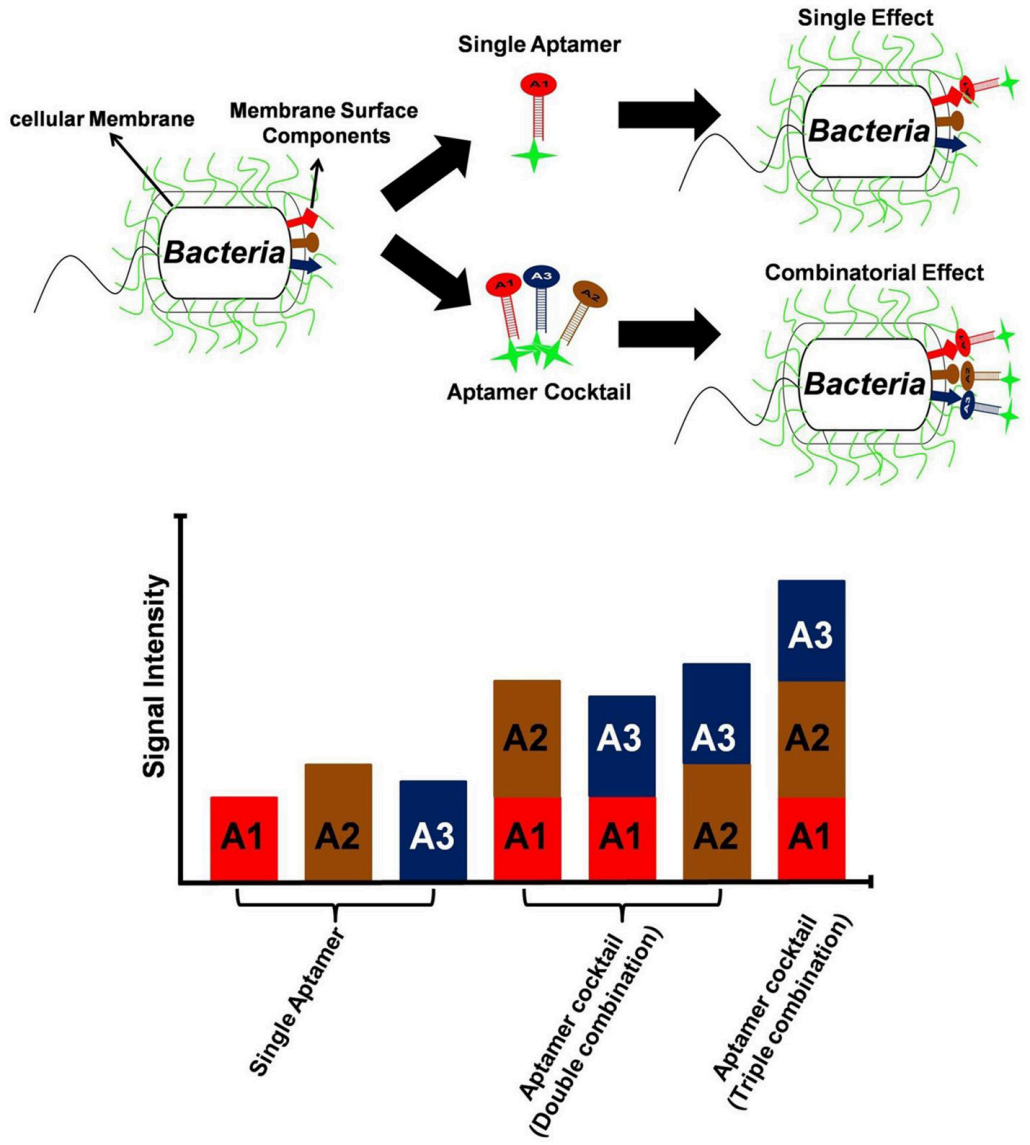
Utility of aptamer cocktail in enhancing signal detection. Schematic concept of signal enhancement by aptamer cocktails for microbial cell detection and increase in fluorescence intensity of bacterial cell suspensions by the interaction with single aptamers or aptamer cocktails.

One clear means to increase aptamer affinity is to add more forces of attraction in the form of more binding to the target. This approach is analogous to the immunology concept of increasing antibody avidity by pooling together multiple monoclonal antibodies or using a polyclonal antiserum. Cao et al. demonstrated for the first time, the superiority of aptamer mixture consisting five high affinity ssDNA aptamers, over single aptamer assays to identify *S. aureus* in pyogenic fluid samples (Cao et al., 2009). This polyclonal aptamer-avidity principle was illustrated by Kim Y. S. et al. (2014), who demonstrated a significant increase in signal strength and an enhanced limit of detection for their *E. coli* aptamer-based assays by utilizing a cocktail of three aptamers versus any of the individual aptamers, (Figure 3). A similar approach is to develop longer multivalent or multidentate aptamers with multiple binding sites for complex targets such as multiple epitopes on protein, bacterial, or other cell surfaces (Vorobyeva et al., 2016). It must however be noted that unlike monovalent aptamers, construction of multivalent aptamer is critically dependent on the linker/scaffold that joins the two aptamers. After all, the length and flexibility of linker will ultimately determine the orientation and position of aptamers with respect to the target and therefore dictate the overall aptamer binding kinetics. Ahmad et al. first described a directed evolution-methodology to obtain high affinity bivalent aptamer against thrombin (Table 1). The authors have fused together two non-competitive thrombin-binding aptamers Bock-15 (that targets fibrinogen-binding exosite) and Tasset-29 (that binds heparin-binding exosite) through a random linker. After five rounds of SELEX, a bi-functional high affinity aptamer was identified with <10 pM K_d_ and over ~200 fold improvement in affinity than monomeric aptamers (Ahmad et al., 2012). This methodology of evolving linker region is highly recommended for generation of multivalent constructs. Hasegawa et al. (2016) have discussed utilizing rational engineering for construction of multivalent aptamers, by joining of aptamer binding motifs. By contrast Bruno (2015b) advocates allowing nature to dictate the length of multivalent aptamers by starting the SELEX process with libraries having longer degenerate or randomized template region. Unlike past aptamers which were generally capped at 100 bases in length, the current routine synthesis of aptamer templates which are up to 200 bases in length is enabling much longer randomized regions leading to tighter binding and greater specificity for these multivalent aptamers (Bruno, 2015b, 2016). Another method for enhancing avidity or overall affinity may be the use of different aptamers attached to the ends of dendrimer strands. Zhang et al. (2015) used this approach for high affinity targeting, imaging and drug delivery to cancer cells and Bruno (2015a) tethered a model aptamer to the 512 amino-terminated strand ends of a generation 7 PAMAM dendrimer and characterized the conjugates by electrophoresis.

### Specificity

In the development of any diagnostic point-of-care test (POCT), it is imperative that the sensory module utilized (aptamer) recognizes only the desired target and should not cross-react with any other non-target molecule present in the sample mixture. This selective binding of aptamer to only the chosen target molecule and no other in the entire sample mixture is defined as the specificity and is necessary to minimize false positive results. Small changes in target specificity can sometimes be achieved, without changing aptamer structure, through mutation of a few bases (Huang and Szostak, 2003). By mutagenesis of three critical nucleotides arginine specific aptamers have been successfully evolved from citrulline aptamers (Yang et al., 1996; Famulok, 1999). Other preceding methods (Table 1) such as XNAs, X-aptamers, AEGIS, engineered multivalent aptamers, and aptamer-conjugated scaffolds are somewhat sophisticated and may not be possible in all laboratories. However, quite often it is sufficient to counter select an aptamer candidate library using related cell species or similar molecules after a few initial rounds of SELEX against the target, to achieve noticeably greater specificity (i.e., via negative selection). This is similar to the immunology concept of adsorbing polyclonal antiserum to remove any non-specific population present that may cross-react with unwanted targets or interferents. Bruno et al. (2011a) were successful in identifying 8 of 100 cloned candidate aptamers which could distinguish minor *E. coli* clone-induced variations in recombinant human growth hormone (hGH) from natural pituitary hGH using this method. High specificity of aptamers is supported by the findings of Chen et al. (2015) who were first to target a single amino acid mutation through aptamers. As more than half of human cancers are associated with p53 mutations, the unique physiologically functional RNA aptamer described by their group could specifically target and rescue p53R175H mutant *in vitro* and xenografts. Occasionally, many authors have used counter-selection steps in SELEX to produce highly specific aptamers that bind bacteria with genus, species, strain, serotype, or even serovar levels of specificity (Bruno et al., 2015; Hamula et al., 2015, 2016). Thus, increasing the time of exposure of aptamer pools to increasing amounts of non-target molecules in successive SELEX rounds encourages selection of only target specific aptamers. But it is important to remember that chemically, oligonucleotide aptamers are polyanions and despite the counter-selection steps in SELEX, they may show varying degrees of attraction toward cationic molecules. This is especially important to consider when the target molecule itself is highly positively charged. In order to minimize selection of charge-based non-specific aptamers, incorporation of polyanionic detergents or surfactants such as dextran sulfate, etc. is highly recommended during SELEX to mask the non-specific electrostatic interactions (Kim K. et al., 2014; Yoon et al., 2015; Bruno, 2017b). Additionally, cation-chelating additives such as EDTA can positively impact selection specificity especially in complex biological milieus that may be high in cations such as Ca^2+^ (Bruno, 2017b).

It is important to note that the primer-binding sites flanking the random region within nucleotides libraries eventually become a part of evolved aptamers as they are necessary for amplification steps in conventional SELEX strategies. These extra-sequences are often suspected to cause non-specific binding thereby leading to loss of valuable oligonucleotides in SELEX counter-selection steps or even interfere with target-aptamer interactions (Pan and Clawson, 2009; Tsao et al., 2017). Additionally, these flanking sequences are sometimes difficult to remove post-SELEX, due to destabilization of a proximal binding motif or the overall aptamer structure. This had lead researchers to explore means of developing aptamers with minimal or no primers (Vater et al., 2003; Jarosch et al., 2006; Pan et al., 2008; Pan and Clawson, 2009; Tsao et al., 2017). However, most of these methods are labor intensive or time consuming with immense dependency on expensive resources. As an alternative, introduction of multiple negative selection steps in SELEX ensures removal of primer-binding domain associated non-specificity to a major extent (Li et al., 2017). Tsao et al. (2017) employed three strategies to enhance the specificity of their aptamers: (i) multiple rounds of negative selection to remove non-target oligonucleotides; (ii) use of primer-free library to minimize interference from non-participating sequences and (iii) enhanced stringency of selection i.e., use of externally applied magnetic field to suppress non-specific events in MARAS aptamer selection.

An often overlooked, but potentially crucial aspect of aptamer specificity is careful initial target or epitope selection. It does little good for specificity to develop high affinity aptamers that bind conserved regions on the target that may span many species or may be shared between related proteins and off-targets, or may even be buried in cell membranes making them inaccessible. Aptamer developers should always take advantage of the rich protein sequence database information and other scientific literature before embarking on aptamer development against proteins. Attempts should always be made to first find unique and accessible peptides or other potential epitope regions of the target for aptamer development.

**Figure 4:**
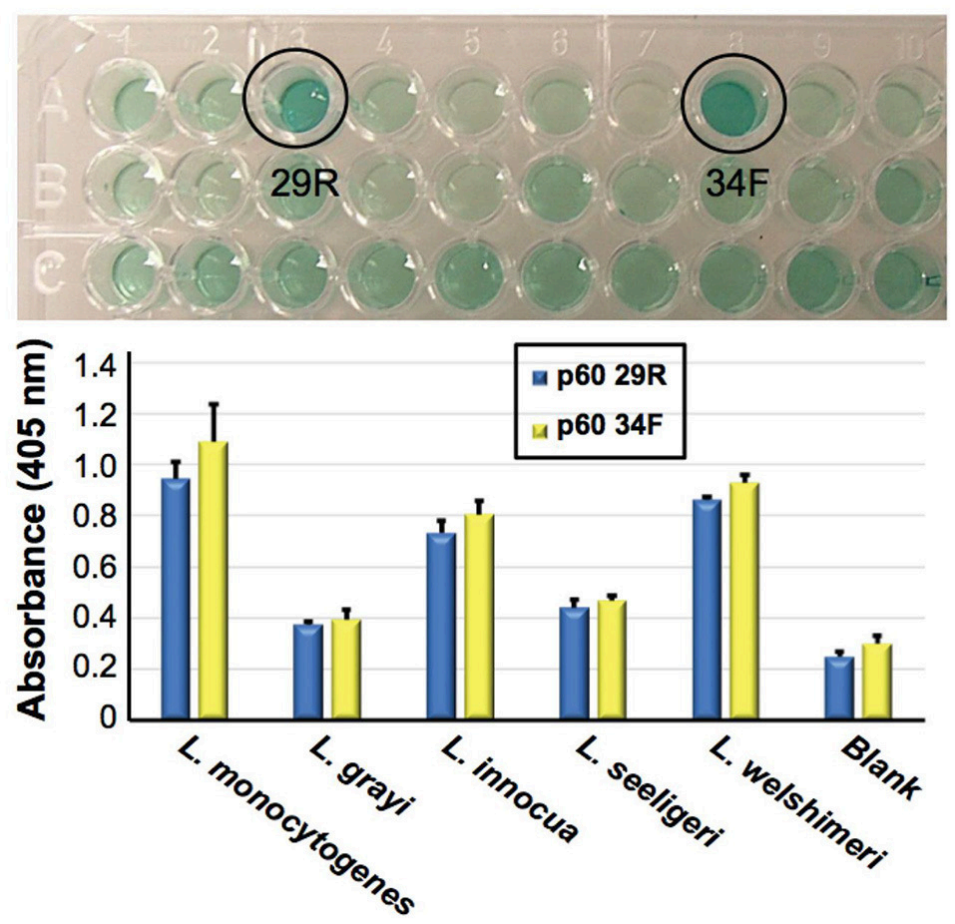
Identification of two candidate aptamers which were selected to bind *Listeria monocytogenes* specific peptide epitope (QQQTAPKAPTE). Top—initial ELISA-like (ELASA) screening assay results, which identified p60 29R and 34F as the best or highest affinity aptamers for binding to the peptide. Bottom—ELASA results for these two aptamers vs. a variety of *Listeria* species. Both aptamers exhibited a preference for binding to immobilized *L. monocyogenes* whole viable cells. Bar heights and error bars represent the Means ± 2 Standard Deviations of six independent ELASA readings (Bruno and Sivils, 2017).

As an example, described is Figure 4, in which Bruno's group has been developing aptamers against the p60 virulence factor of *Listeria* species (Bubert et al., 1994). Previous reports from Bruno's group demonstrated aptamer development against listeriolysin, which was specific only to the *Listeria* genus level (Bruno et al., 2015). Recently however Bruno and Sivils (2017) have targeted the peptide region QQQTAPKAPTE unique to the p60 of *Listeria monocytogenes* (Bubert et al., 1994; Coutu et al., 2014) and have produced two new aptamer candidates designated p60 29R and 34F (Figure 4, Top) which appear to prefer binding to *L. monocytogenes* whole cells by ELASA (Figure 4, Bottom).

### Selectivity

**Figure 5:**
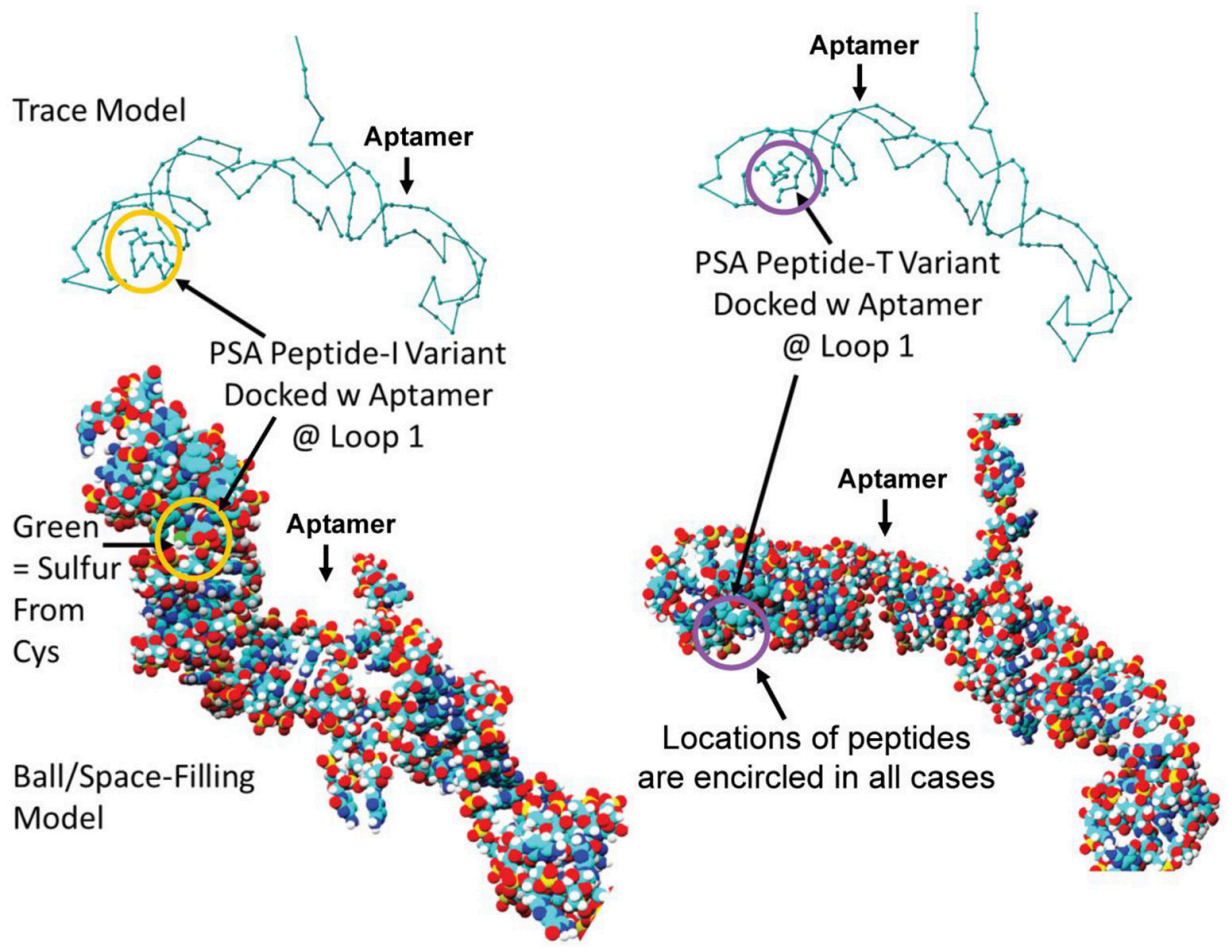
Results of initial YASARA-based 3-D molecular modeling to identify where the PSA variant isoleucine (I) vs. threonine (T) peptide regions bound to the candidate aptamer. Top—trace models of the aptamer docked with the two variant PSA peptides reveal binding to the same loop structure. Bottom−3-D space-filling ball models of the same binding events. Peptides are encircled in each case to distinguish their locations within the loop structure designated Loop 1 in the docked aptamer-ligand complexes.

**Figure 6:**
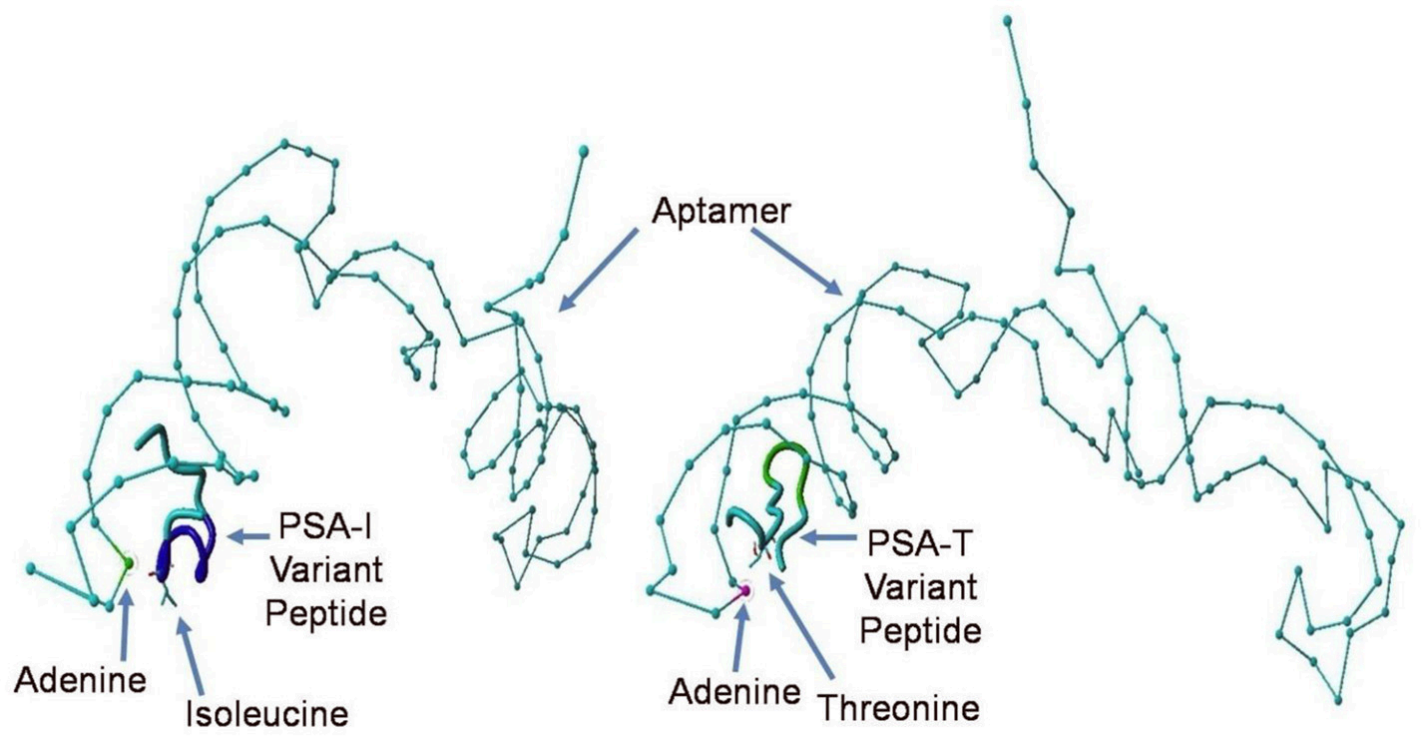
Close up views of the top PSA aptamer binding to both, the I and T peptide variants. This tube structure docking analysis revealed the presence of a common proximal adenine in the aptamer binding site as indicated by arrows directly across from the indicated isoleucine (I) or threonine (T), which could be replaced by a diaminopurine (DAP) base to alter binding in the region and potentially discriminate the I- vs. T-PSA peptide variants.

**Figure 7:**
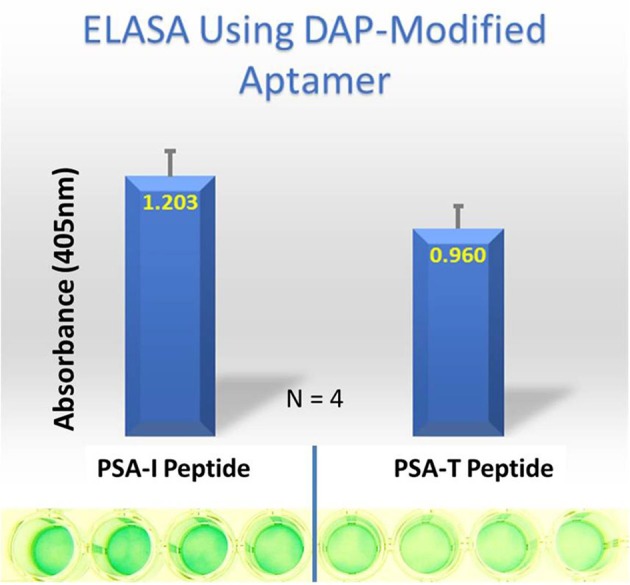
ELASA results demonstrating differential binding of the DAP-modified aptamer to the PSA variant peptides. The DAP-modified aptamer demonstrated ~ 20% greater affinity for the PSA-isoleucine (I) vs. the PSA-threonine (T) variant. Bar heights and error bars represent the Means ± 2 Standard Deviations of four independent enzyme-linked (ELASA) absorbance at 405 nm.

Despite all efforts to ensure a clear high degree of specificity and high affinity binding of the generated aptamers to the desired target, aptamers may still demonstrate some level of binding, albeit lesser, to non-cognate molecules. This may be due to charge-based attraction or presence of a sequence, epitope, or domain similar to the target. In other words, while the generated aptamer may not be completely specific, it may be selective for binding maximally to the desired target and several fold lesser to non-target molecules. Since achieving 100% specificity may not be possible, efforts are ensued to generate aptamers that are as selective as possible. Application of aptamers in sandwich format or use of multivalent aptamers enhances the assay specificity several fold, even more than the specificity of the individual aptamers used. In another approach, selective aptamers may be deliberately required to discriminate between genetic variants of the same target. As an example, toward development of a better prostate specific antigen (PSA) test, Bruno investigated a PSA peptide region wherein isoleucine (I) and threonine (T) genetic variants exist at position 179 in the full PSA protein (Kote-Jarai et al., 2011; Sullivan et al., 2015). The current highest affinity aptamer candidate cannot discriminate the I and T variants (data not shown). Thus, Bruno used 3-D YASARA docking models as illustrated in Figures 5 and 6 to unveil the insight that a particular adenine in the candidate aptamer is proximal to the I and T locations in the PSA peptides (Albada et al., 2015; Rhinehardt et al., 2015). Bruno then reasoned that substitution of a DAP for the adenine proximal to I and/or T would either lead to an additional hydrogen bond (strong attractive force) or result in an additional repulsive force between the extra amine group's electron pair in DAP and the highly electronegative polarized oxygen in the hydroxyl group of threonine, thus providing a means for discrimination and greater selectivity. While this approach only produced about a 20% greater binding of the modified aptamer to the PSA I-variant by ELASA (Figure 7), this suggested that the aptamer-peptide binding was perturbed in that region by an increased repulsive force between DAP and threonine of the PSA T-variant. Thus, Bruno is continuing to investigate other exotic base insertions to the candidate PSA aptamer in and around the DAP insertion site through synthesis by Integrated DNA Technologies, Inc. (Coralville, IA, USA) using their current manual of available exotic bases. Perhaps other modified or unnatural bases can produce a >20% difference in PSA variant binding, thus leading to a better and more discriminatory PSA test capable of detecting more patients with aggressive prostate cancer in need of earlier cancer treatment (Kote-Jarai et al., 2011; Sullivan et al., 2015). As another example, neurotransmitters and small biomolecules involved in signaling are often structurally related. This necessitates the need for their selective sensing in the same brain region. The utility of DNA aptamers coupled to Field-effect transistors (FETs) has been demonstrated by Nakatsuka and Andrews for the selective and simultaneous detection of structurally similar dopamine and norepinephrine neurotransmitters (Nakatsuka and Andrews, 2017).

## Enhancing aptamer assay parameters

In a recent review, Sharma et al. (2017) have summarized the careful steps involved in successful aptamer and related assay development. Basically, every POCT utilizes two critical components: a sensor and a detection module. Since a diagnostic platform essentially measures the sensor element, which indirectly correlates with the presence/amount of analyte in the sample; the success of any POCT is therefore dependent upon the performance of both the components. On the one hand, enhancing the affinity, avidity and specificity of aptamers that directly bind the target would certainly enhance the specificity and sensitivity of the test; greater test sensitivity is also dependent upon the nature of the linker system used.

### Assay sensitivity or limit of detection

While anti-digoxigenin antibodies that bind digoxigenin-labeled aptamers provide a comparably high affinity linkage (Bruno, 2014); the classic biotin-streptavidin system with a K_d_ of femtomolar range maximizes assay sensitivity (Kendall et al., 1983). Similarly, the detection modality can greatly enhance sensitivity by providing a high signal-to-noise ratio (SNR; low background signal). Current aptamer-based electrochemical detection methods are providing ultrasensitive detection via high signal to noise ratios (Hernandez and Ozalp, 2012). Similarly, aptamer electro-chemiluminescence (ECL) assay systems have demonstrated sub-picogram/ml detection limits (Bruno et al., 2014). Aptamer capturing approaches also enhance the assay sensitivity by concentrating the analyte that may otherwise be present in low amounts in the sample. Using this approach and utilizing an enzyme specific fluorescent substrate, Zhao et al. (2011) detected as low as 2 fM human alpha thrombin and 100 fM human neutrophil elastase (HNL) in serum samples. The pros and cons of various aptasensors and the analyte detection limits with the development time and cost, has been well summarized by Gopinath et al. (2016) as they are critical for the development of appropriate POCTs. While electrochemical, SPR or fluorescence based methods provide maximal assay sensitivity; they are costly and require sophisticated instruments and dedicated set-up that is not always feasible. Nanoparticles functionalized with aptamers have provided a sensitive alternative for development of POCT. For example, in a lateral flow strip format, aptamer functionalized Gold-nanoparticles (AuNPs) have shown unequivocal visual detection of down to 2.5 nM thrombin (signal/noise = 3), providing superior sensitivity and specificity relative to conventional antibody-based assays (Xu et al., 2009). A FRET-based aptasensor was designed by Meng et al. (2016) using functionalized AuNPs as energy acceptors; their assay showed no-cross reactivity to other nucleotides and could specifically detect ATP as low as 15.2 nmol/L.

### Assay specificity

Since achieving 100% aptamer specificity is not always possible, maintaining an intricate balance between the specificity and sensitivity of the downstream aptamer-assay is required. A sandwich-type bioassay overcomes the limitation of a single-aptamer assay and shows an overall multifold enhanced sensitivity as well as specificity. This is in particular very useful when sample volume is not a limitation. Seo and Gu (2017) have very thoroughly compared various sensing modalities and platforms (colorimetry, electrochemistry, surface plasmon resonance, impedance on an electrode) of sandwich-type aptasensors and their influence on the limit of detection of analytes in samples. Finally, aptamer assay specificity can be remarkably enhanced by post-experiment principle component analysis and Bayesian classification or other advanced statistical analyses which place detected analytes in their most likely cluster groups based on bivariate or multivariate graphing and analyses (Ostroff et al., 2010; Zhong et al., 2011; He et al., 2013).

Achieving high sensitivity and specificity is the essential core, upon which the success of every POCT rests. Combining high sensitivity and specificity of the target-sensing aptamers, together with the high sensitivity and specificity of the detection module provides highest assay efficiency, crucial in development of POCTs. As summarized previously, readers may introduce feasible innovations in SELEX starting with the design of initial random library, modification of selection conditions, introduction of counter-selection steps and also incorporate post-SELEX optimizations, to enhance the affinity, avidity, selectivity, and specificity of aptamers; to explicitly enhance the overall assay sensitivity and specificity. Due to the *in vitro* mode of selection and removal of dependency on host animals and therefore batch-to-batch variation; aptamers having high affinity and specificity can be chosen with ease, unlike the antibodies. It is not surprising if aptamers may someday usurp antibodies from the existing diagnostic and therapeutic applications, due to their clear advantages. Further, accuracy of aptamer assays can easily be enhanced multifold in combination with sensitive linker systems such as Gold-nanoparticles (AuNPs), electrochemical detection, and aptamer-capturing approaches, to achieve ultrasensitive levels of target detection. Therefore, keeping in mind the cost, time consumption, feasibility, dependency on specific instruments etc., the reader may enhance aptamer performance and/or combine aptamers with appropriate linker-formats to develop assays of high sensitivity and specificity.

## Success studies of aptamers in diagnostics

As summarized by Pfeiffer and Mayer (2016) numerous companies are involved in commercialization of aptamers across the globe. Some of the successful examples are described below:

### NeoVentures OTA-sense system

Key to development of a diagnostic assay is: (i) cost of production, (ii) consistent sensitivity across batches, (iii) Sensitivity in compliance with regulatory standards, and (iv) advantage over existing products. In compliance with these directives Dr. Gregory Penner of NeoVentures developed an aptamer-based Ochratoxin A (OTA) detection kit for commercial application in agro-food industry (Penner, 2012). OTA is a mycotoxin that not only impairs blood coagulation (Wu et al., 2018), but is also a potential carcinogen (Pfohl-Leszkowicz and Manderville, 2007) produced by fungus in contaminated food products. Pioneering work by Penner's group evolved highly sensitive (~200 nM K_d_) and specific 36 nt aptamer with potential to detect ppb quantities of OTA in naturally contaminated wheat samples (Cruz-Aguado and Penner, 2008). While translating the application of these aptamers to develop sensors, the OTA-Sensing System (NeoVentures) was designed (http://neoventures.ca/products/mycotoxin-testing/). In this system, sample is first ground, extracted with four volumes of 60% acetonitrile. The extract is thereafter diluted with binding buffer to have uniform pH followed by filtration through glass wool, after which it is loaded onto the OTA-sense affinity column. OTA toxin if present in the sample is retained on the OTA-aptamer immobilized resins on column. The bound toxin is then eluted with cation free buffer and combined with detection solution containing free aptamer and terbium. Terbium creates a cation bridge between OTA and aptamer, which enhances the fluorescence of Terbium (380 nm Excitation−540 nm Emission). The free aptamer/terbium detection step provides the targeted market advantage, as it is adaptable to a comfortable 96-well plate format with high sensitivity. The kit format also eliminates the cost of running calibration standards providing immense advantage over traditional ELISAs. The OTA-Sense System has been extended to include all alcoholic beverages, including beer and white and red wine. NeoVentures is actively pursuing to develop similar applications to detect other major mycotoxins including aflatoxin, zearalenone, fumonisin and deoxynivalenol. Although the OTA-aptamer was generated through conventional SELEX strategy, the success of OTA-Sense System can be attributed to the beneficial combination of high affinity and specificity of aptamers with a reliable and sensitive detection system (Penner, 2012).

### Somalogic (Boulder CO, USA)

Providing the enormous advantages of (i) chemical diversity, (ii) high affinity (nano to picomolar range K_d_), (iii) specificity, especially for those targets that fail conventional SELEX (Gold et al., 2010), it is unsurprising to find SOMAmers and SOMAScan aptamer array platforms to be successfully established (http://somalogic.com/technology/our-platform/). Using modified aptamers with multiplexed-proteomics technology, high throughput screening of multiple biomarkers is possible in limited volumes of samples (150 μl). Marketed as SOMAmer Reagents by SomaLogic, applications are widely beneficial to the proteomics and diagnostics market with the current capability to detect >1,310 human proteins in sub-pg levels in body fluids (Lollo et al., 2014; Webber et al., 2014). Further, compatibility to be customized to routine lab technologies such as ELISA, Mass Spectrometry, flowcytometry, histology and cell microscopy, increments its translational value multifold.

### APOLLOMER^TM^ probes (CibusDx, Inc)

CibusDx, Inc. acquired Pronucleotein and licensed ApolloDx's aptamer based diagnostic platform of food safety testing for commercialization, (http://apollodx.com/apollodx-licenses-technology-food-safety/). The ApolloDx's technology is based on test strips with proprietary aptamer-based APOLLOMER^TM^ probes, that bind specific targets of foodborne and waterborne pathogens, biological, and chemical toxins as well as bacterial cell capsules, parasites and virus present in the test sample in <30 min. A handheld electrochemical analyzer compatible with smartphone/tablet, detects the binding events on strip, making it convenient for use in outdoor settings. The low-cost, combined sensitivity and reliability of aptamers and electrochemical sensor, contributes to the success of this efficient easy-to-use POCT.

### Sekisui diagnostics, GmbH

Thrombin is an essential serine protease, that plays a crucial role in the blood coagulation pathway. Therefore, estimation of active thrombin levels is critical to identify patients predisposed to bleeding or thromboembolic complications. Routine estimation protocols, either estimate prothrombin fragment F1,2 released after proteolytic activation of thrombin; or the inactivated thrombin-antithrombin-complexes (TAT). Since both the components are unevenly distributed in circulation, neither protocols accurately estimate the true *in vivo* levels of active thrombin (Merlini and Ardissino, 1995). In this regard, a novel oligonucleotide-based enzyme capture methodology was designed by Müller et al. (2011) to accurately estimate blood thrombin levels. Marketed under the name of OLIGOBIND® Thrombin activity assay by Sekisui Diagnostics (https://www.sekisuidiagnostics.com/products/722-thrombin-activity-assay), the kit accurately measures active thrombin levels through an aptamer based enzyme-capture fluorescent assay (Königsbrügge et al., 2017). In the developed assay, plasma samples are added to microwell plates pre-coated with thrombin specific aptamers. After optimized incubation time and washes, thrombin bound to coated aptamers is measured by addition of a fluorogenic substrate (Excitation−360 nm; Emission−460 nm). Obtained sample values are extrapolated to a standard curve to quantify up to picomolar concentrations of thrombin in samples. The success of this aptamer based-diagnostic kit can be attributed to the utilization of ultrasensitive HD1-22 bivalent aptamer that demonstrates ~100-folds higher selectivity for thrombin (than prothrombin) along with sub-nanomolar affinity (Müller et al., 2011). Further the choice of fluorescent substrate for detection, enhanced the kit sensitivity multifold.

Based on the same principle Müller et al. (2012) designed a activated protein C (APC) specific aptamer-based enzyme capture assay, marketed under the name OLIGOBIND^®;^ APC activity assay by Sekisui Diagnostics, GmbH, (https://www.sekisuidiagnostics.com/products/724-apc-activity-assay).

## Conclusions and future perspectives

The field has recently celebrated 25 years of aptamer technology discovery aptamers have evinced an impressive growth trajectory as powerful diagnostic reagents. However, in comparison to antibody this technology still appears to be in infancy. With the advent of Xeno nucleic acids or extended genetic codes, multidentate aptamers and modern SELEX technologies second generation aptamers are ready to revolutionize the diagnostics industry. Using the aforementioned modifications and one-step isolation methods to rationally tailor the aptamers of choice, a panel of aptamers with even greater affinity, avidity, specificity, selectivity and stability can be designed. Using this new class of aptamers an array of ultrasensitive invincible aptamers can be developed that can detect the analyte in a highly sensitive and specific manner. If required, limit of detection of an aptamer can further be improved by adapting to a more sensitive detection platform such as electrochemical sensing etc., that may lead to a successful commercial product for diagnostics applications. Indeed, some of the successful aptamer products highlighted in the current article are already paving their way forward in diagnostics industry and forecasting the bright future of aptamer technology in biomedical industry. However, it is first necessary for global diagnostics manufacturers who have supported and invested substantial amounts of capital over the years in antibody development; to overcome their hesitation and invest in aptamer diagnostics platforms. By utilizing the rational engineering approach that tailors affinity, specificity and selectivity of aptamers, a new wave of diagnostic reagents can be created especially for those applications where antibodies perform sub-optimally or fail to work.

## Author contributions

TKS and JGB: conceived the idea; PK, AD, TSK, JGB and WCC: wrote and proofread the manuscript. All authors read and approved the manuscript.

### Conflict of interest statement

JGB is an employee of OTC Biotechnologies, LLC. He owns 8% share of OTC Biotechnologies, LLC, which is an aptamer company. TKS owns the 87% stake in AptaBharat Innovation Pvt. Ltd. India. The other authors declare that the research was conducted in the absence of any commercial or financial relationships that could be construed as a potential conflict of interest. The handling Editor declared a past co-authorship with one of the authors WCC.
